# Protein supplements consumption: a comparative study between the city centre and the suburbs of Palermo, Italy

**DOI:** 10.1186/2052-1847-6-29

**Published:** 2014-07-12

**Authors:** Antonino Bianco, Caterina Mammina, Ewan Thomas, Francesco Ciulla, Umberto Pupella, Francesco Gagliardo, Marianna Bellafiore, Giuseppe Battaglia, Antonio Paoli, Antonio Palma

**Affiliations:** 1 Sport and Exercises Research Unit, University of Palermo, Via Eleonora Duse, 2-90146 Palermo, Italy; 2 “G. D’Alessandro” Department of Sciences for Health Promotion and Mother-Child Care, University of Palermo, Via del Vespro, 127, 90129 Palermo, Italy; 3 Department of Biomedical Science, University of Padua, Via Manzolo, 3, 35131 Padova, Italy

**Keywords:** Protein consumption, Dietary supplements, Demographic assessment

## Abstract

**Background:**

Protein supplements are extensively used among commercial gym users and athletes. Although demographic information amongst such consumption has been poorly investigated. The aim of this study is to compare protein supplement consumption in commercial gym users, both from the city centre and the suburbs of Palermo, Italy.

**Methods:**

A face-to-face questionnaire has been administered to 561 subjects. 207 from the city centre (CC) and 354 from the suburbs (SB) of Palermo, Italy. Protein supplement consumption, frequency of use and association with other dietary supplements has been investigated. Subsequently frequency distribution has been used for demographic assessment.

**Results:**

30% of commercial gym users from the CC and 28.8% from the SB use protein supplements. The majority of these subjects are men (69.5% from the CC and 93.1 from the SB). Frequency of use differs from the two areas showing a mode of 7 days per week consumption for the CC while a mode of 5 days per week consumption is showed for the SB. Interestingly, the association between protein supplements and other dietary supplements also differs between CC and SB. A combined use between proteins, amino acids and creatine is showed in the CC whilst proteins and amino acids or creatine are used alone in the SB.

**Conclusions:**

A considerable number of commercial gym users take protein supplements alone or combined (mainly creatine and amino-acids). Demographic differences are shown between frequency of use and combined use of other dietary supplements. We emphasize on the importance of the dissemination of scientifically based information about supplementation in this environment.

## Background

Supplements are consumed for a variety of reasons. Many active individuals use supplements for a variety of reasons. Muscle gain, strength improvement, prevent future diseases or illnesses and improve performance in sport are some of the main [1,2]. It has been also shown by a variety of studies that people have a number of different opinions about the usage of supplements [3-12].

Kaufman et al. [13] found that older people were more likely to take multivitamin and mineral supplements, while younger people were more likely to take creatine. The choice of supplements also depends on the typology of the exercise program [8] and the type of sport [3].

In particular regarding supplement consumption in commercial gym users, proteins are the most consumed as shown by Scofield et al. [3], Morrison et al. [8] and Bianco et al. [14-16]. As shown by Pechey et al. [17], socioeconomic status is another factor influencing the quality of food intake, highlighting that low socioeconomic status people usually purchase a greater proportion of unhealthy foods and beverages. Conversely, high socioeconomic status people purchase greater proportions of fibres, proteins and total sugars, and smaller proportions of sodium. Duran et al. also found differences between neighbourhoods in regard the quality of food intake [18]. A restricted number of studies, though, have investigated this specific topic and is not in our knowledge if dietary supplement consumption in particular protein based supplement consumption differs between the city centre (CC) and the suburbs (SB).

The aim of this study, therefore, is to compare the utilization of protein supplements, assess their frequency of use and their consumption in combination with other dietary supplements. Subsequently compare commercial gym adepts from the CC and the SB of Palermo, Italy.

## Methods

### Participants

Permissions to conduct a survey were obtained from the managers of twelve fitness centres located in the suburbs of Palermo, in 2013. The fitness centres were identified using a database of the CONI register (National Olympic Committee Register for Sport and Fitness Associations). Using the database of the fitness centres, a number of 1200 people (20% of the total number), have been randomly selected as potential participants. The inclusion criteria of the study were: 1) fitness/gym attendees taking part in strength training courses 2) not taking part in any aerobic activity (such as aerobic, spinning, step, circuit training, endurance and cardiovascular programs). Such inclusion criteria were set in order to understand the frequency consumption of protein supplements in people involved in resistance training. No obese people were involved in the study (Subjects who’s BMI >30 were particularly muscular). On the basis of these inclusion/exclusion criteria, a total of 354 participants were involved for the investigation. These subjects were then compared with those from our previous study (207) [14].

### Questionnaire procedure

In order to evaluate supplements use, dietary behaviour and other related information’s, the questionnaire adopted in 2011 by Bianco et al. was used [14-16] (Additional file 1). The questionnaire was administered in commercial gyms of the suburbs of Palermo, Italy. Easy definitions of the supplements were provided to the participants (Common and commercial names of products or substances included within the definition of supplement: product intended to supplement the diet that contains one or more dietary ingredients) [19]. Completion of the questionnaire was considered the implied consent to participate in the study. According to the Italian regulations, ethical approval was not required for this study. The questionnaire was administered by the same investigator using the face-to-face interview method during a period of six months.

### Data analysis

Data analysis was performed using EpiInfo software version 7.0 (CDC, Atlanta, GA, US) and Statistica version 8.0 software for Windows (Tulsa, OK, US). The descriptive analysis was performed by calculating the means, standard deviations and frequencies. Differences were assessed by a one-way ANOVA test. The associations between the variables under examination were evaluated using contingency tables. Statistical significance was set at *P* values ≤ 0.05.

## Results

### Demographic results

561 questionnaires were used for comparisons (354 from the suburbs and 207 from the city centre) representing 434 male and 137 female. These were 80 females and 127 male from the CC and 47 females and 307 male from the SB. The majority of subjects in both groups were age ranging between 18 and 30 years, as shown in Tables 1 and 2. Moreover, the frequency of supplement consumption amongst both groups were around 30% of total (30% CC and 28.8 SB), though a significant gender difference between the two areas is evident.

**Table 1:** Anthropometric characteristics of participants stratified by CC and SB

	***Subjects***	
	***Number***	***Percentage***
**Age (yr)**		
< 18	62	11%
18-30	314	56%
> 30	185	33%
Mean (SD)	28,2 ± 10 yrs	
**Gender**		
Female	127	22.6%
Male	434	77.4%
**Body mass index**		
< 25 kg/m^2^	385	68.63%
25 ≤ 30 kg/m^2^	150	26.74%
≥ 30 kg/m^2^	26	4.63%

**Table 2:** **Anthropometric characteristics of all participants**, **Palermo**, **Italy**

	***City centre (207)***	***Suburbs (354)***
**Age (yr)**		
< 18	11.1%	11%
18-30	65.7%	50.3%
> 30	23.2%	38.7%
**Gender**		
Female	38.65%	13.3%
Male	61.35%	86.7%
**Body mass index**		
< 25 kg/m^2^	71.9%	66.7%
25 ≤ 30 kg/m^2^	24.6%	27.9%
≥ 30 kg/m^2^	3.5%	5.4%

The CC gym users who undergo protein supplement consumption were 69.5% of man vs. 30.5% of women (*p* < 0.001) while in the SB this percentage significantly raises in man showing 93.1% vs. 6.9 for women (*p* < 0.001).

### Supplement consumption

Frequencies of consumption are shown in Tables 3 and 4. The major results indicate a higher weekly frequency of protein supplement consumption in the CC (Mode of 7 times per week) respect to the SB (Mode of 5 times per week). Other noteworthy observation is that in both groups a frequency of protein consumption of 3 times per week is highly expressed. See Tables 3 and 4 for percentages.

**Table 3:** **Frequency and type of dietary supplements used by all participants**, **Palermo**, **Italy**

	**Subjects**	
	**Number**	**Percentage**
**Supplements use**		
No	397	70.8%
Yes	164	29.2%
**Supplement users (164)**		
Male	138	84.1%
Female	26	15.9%
**Frequency of use (164)**		
1 time per wk	9	5.5%
2 time per wk	9	5.5%
3 time per wk	46	28%
4 time per wk	18	11%
5 time per wk	59	36%
6 time per wk	2	1.2%
7 time per wk	21	12.8%
Other supplements (107)*,		
Vitamins	8	7.5%
Creatine	15	14%
Amino acids	42	39.2%
Creatine + Amino acids	31	29%
Vitamins + Amino acids	2	1.9%
Creatine + Vitamins + Amino acids	9	8.4%

*Other supplements in association with protein supplements.

One subject has declared the use of anabolic steroids.

**Table 4:** **Percentage and type of dietary supplements used by all participants**, **Palermo**, **Italy**

	**Subjects**	
	**City centre (207)**	**Suburbs (354)**
**Supplements use**		
No	70%	71.2%
Yes	30% (62)	28.8% (102)
**Supplement users (Yes)**		
Male	69.5%	93.1%
Female	30.5%	6.9%
**Frequency of use**		
1 time per wk	12.9%	1%
2 time per wk	8.1%	3.9%
3 time per wk	21.0%	32.3%
4 time per wk	17.7%	6.9%
5 time per wk	14.5%	49%
6 time per wk	1.6%	1%
7 time per wk	24.2%	5.9%
Other supplements*,		
Vitamins	4.8%	11.1%
Creatine	6.4%	24.4%
Amino acids	25.9%	57.9%
Creatine + Amino acids	48.4%	2.2%
Vitamins + Amino acids	1.6%	2.2%
Creatine + Vitamins + Amino acids	12.9%	2.2%

*Other supplements in association with protein supplements.

One subject from a suburb area has declared the use of anabolic steroids.

### Association with other supplements

The association between protein supplements and other dietary supplements differs between the CC and the SB. It is shown that the CC group frequently assumes protein supplements in association with creatine and amino acids (48.8% of total), or proteins and creatine alone (6.4% of total) and proteins and amino acids alone (25.9% of total). In the SB the association between protein supplements, creatine and amino acids collapses to 2.2% of total, while the intake of protein supplements and creatine alone raises to 24.4% and protein supplements and amino acids alone to 57.9%. Other associations are shown in Tables 3 and 4.

### Source of information about use of supplements

**Figure 1:**
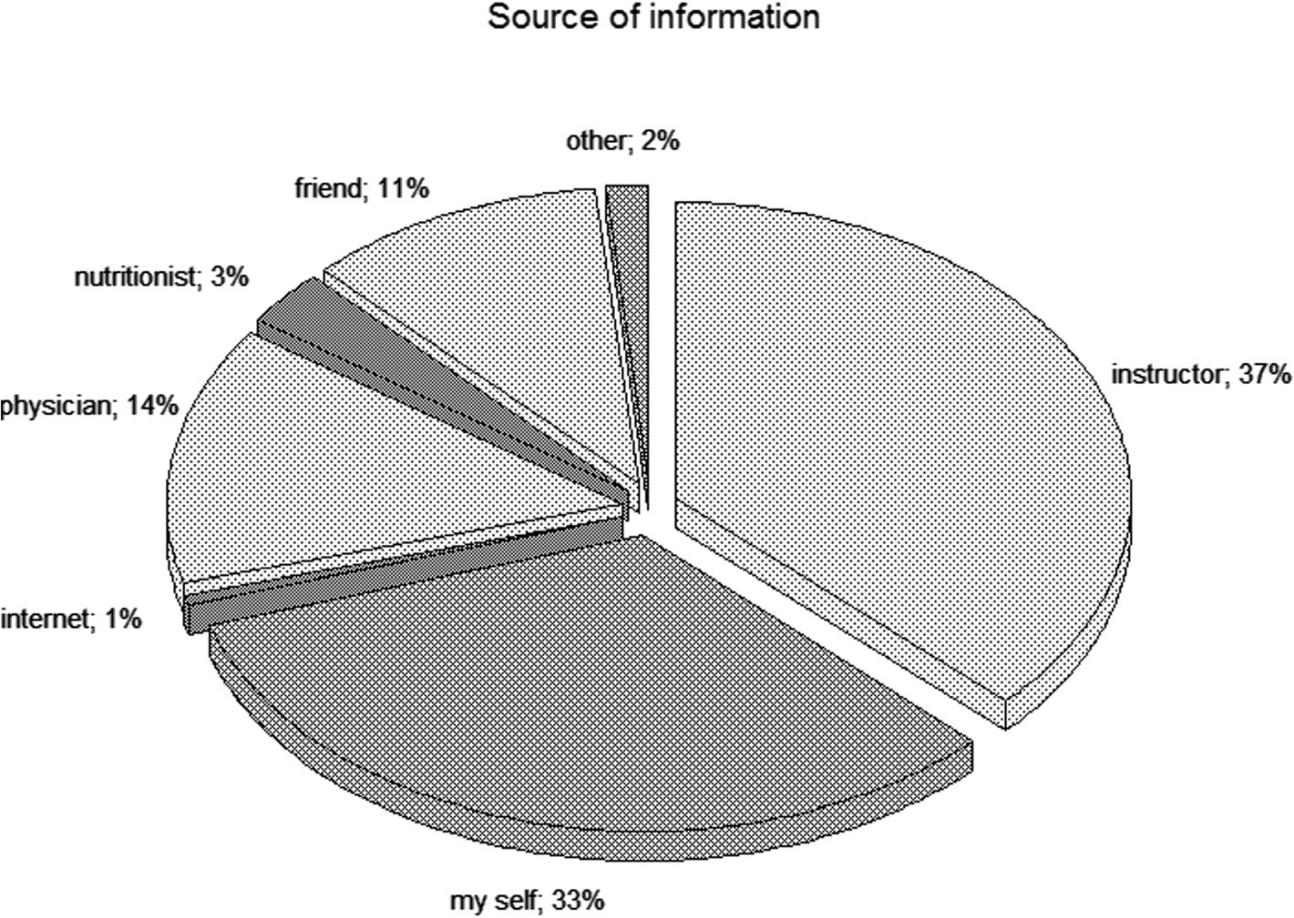
**Source of information about use of supplements.** Distribution of source of information amongst users.

When examining the source of information, the majority of subjects (37.0%) appeared to rely on the gym instructors’ guideline/advice, and on their own knowledge (33.0%) in order to star protein supplement consumption. Only 14.0% of the participants consulted a physician and 3.0% a nutritionist. Whilst, 11.0% of the participants started after friends advice (Figure 1).

## Discussion

The comparison between the current and our previous study has shown a similar age range between participants with a higher prevalence from 18 to 30 years of age. There are no differences in percentage between the first [14] and the current study amongst the consumption of protein supplements (30% CC and 28.8% SB). Both groups highlight that males are more prone to the use of protein supplements than female with differences between the CC and the SB [3,8,20].

Goston and Correia [20] have found that the use of supplements in Brazil, was associated with the people who needed them less, since their diet appeared concurrently to be good or excellent. A similar observation has been described by Conner et al. [21] and Millen et al. [22]. Many authors suggest that athletes need extra proteins in their diet as food or as supplements [23-27], however regular gym attendees do not need these extra supplements [18,24,26].

Other outcome regards the sources of information. Fitness instructors are the major (37%) followed by users own beliefs (33%). It is important to underline that physicians and nutritionist have been poorly consulted (17.0% vs. 3.0%), whereas in Morrison et al. [8] and Goston and Correia’s studies [20] the relative proportion is as high as 30.0%. Further result comes from the frequency of consumption that has a prevalence of 5 days per week in the SB against 7 found in our previous study, data in line with Goston and Correia [20]. The majority of subjects that reported protein supplement consumption also declared a combination of such products with other dietary supplements. The most frequent association was between proteins and amino acids (57.9%) and secondly between proteins and creatine (24, 4%). Interestingly even though in our previous study the association between protein supplements and other dietary supplements was present, this proportion was considerably different (Amino acids 25.9%, creatine 6.4%). The sample size could be considered a limit for the study. Though, the inclusion/exclusion criterion allows us to consider only strength and conditioning adepts. Notwithstanding such condition most of the studies we found, have reported similar sample sizes [8,20]. This might be related to the difficulties to deal with managers and fitness adepts. In order to overcome these difficulties and to increase the sample size, a project named Protein Project (PP) (http://www.proteinproject.com) is currently involving five European Universities, the Italian National Olympic Committee (CONI) and the Grafts Hellas Fitness Company (Greece) into epidemiological and demographic surveys.

## Conclusions

In line with other studies, our results show protein supplement consumption around 30% with higher consumptions in man vs. women. High associations between protein supplements and other dietary supplements, especially creatine and amino acids were shown with frequency differences between the CC and the SB.

As previously showed, we concluded that gym adepts that consume protein supplements were not aware of objective recommendations of protein intake. They have may perceived their needs to be excessively high. It is generally accepted that athletes have increased protein needs due to the intense physical activity they undergo. Though, the position statement of the International Society of Sports Nutrition states that exercising individuals’ protein needs are between 1.4 and 2.0 g/kg/day, depending upon mode and intensity of exercise, quality of protein, and status of total calorie and carbohydrate intake. General population attending commercial gyms usually has less workload than athletes. This underlines that their daily protein intake should be in line or lower than athletes guidelines. The reasons for which there are these differences between CC and SB are still not entirely clear. In order to overcome such lack of information, questionnaires are currently being administered (PP).

## Competing interests

The authors declare that they have no competing interests.

## Authors’ contributions

All authors have effectively contributed to this work in its different production stages. All authors have read and approved the final manuscript.

## Pre-publication history

The pre-publication history for this paper can be accessed here:

http://www.biomedcentral.com/2052-1847/6/29/prepub

## Supplementary Material

Additional file 1 **Protein Project questionnaire adopted by Bianco et al. **[15]**.** Click here for file
